# Implications of Extracellular Matrix Production by Adipose Tissue-Derived Stem Cells for Development of Wound Healing Therapies

**DOI:** 10.3390/ijms18061167

**Published:** 2017-05-31

**Authors:** Kathrine Hyldig, Simone Riis, Cristian Pablo Pennisi, Vladimir Zachar, Trine Fink

**Affiliations:** Laboratory for Stem Cell Research, Department of Health Science and Technology, Aalborg University, 9220 Aalborg, Denmark; kha12@student.aau.dk (K.H.); sriis@hst.aau.dk (S.R.); cpennisi@hst.aau.dk (C.P.P.); vlaz@hst.aau.dk (V.Z.)

**Keywords:** adipose stem cells, ASCs, extracellular matrix, wound healing

## Abstract

The synthesis and deposition of extracellular matrix (ECM) plays an important role in the healing of acute and chronic wounds. Consequently, the use of ECM as treatment for chronic wounds has been of special interest—both in terms of inducing ECM production by resident cells and applying ex vivo produced ECM. For these purposes, using adipose tissue-derived stem cells (ASCs) could be of use. ASCs are recognized to promote wound healing of otherwise chronic wounds, possibly through the reduction of inflammation, induction of angiogenesis, and promotion of fibroblast and keratinocyte growth. However, little is known regarding the importance of ASC-produced ECM for wound healing. In this review, we describe the importance of ECM for wound healing, and how ECM production by ASCs may be exploited in developing new therapies for the treatment of chronic wounds.

## 1. Introduction

Wound healing is a dynamic and well-orchestrated process with both molecular and cellular events. When for some reason the wound healing process is perturbed, the wounds may become chronic, with concomitant alterations in the microenvironment leading to prolonged inflammation, ischemia, dysfunctional extracellular matrix (ECM), and lack of re-epithelialization [1]. Traditional wound healing therapies are often not sufficient, so there is considerable interest in developing novel more efficient therapies. Among the novel strategies that are being explored, the use of adipose tissue-derived stem/stromal cells (ASCs) appears to be very promising, judging from animal studies [2] and early clinical studies [3]. The ASCs are derived from the so-called stromal vascular fraction of adipose tissue [4,5], which is a rather heterogeneous population. However, after expansion for just a few passages, the ASCs converge towards a common phenotype comprised of fewer, perhaps functionally distinct subtypes [6,7].

While it is still not clear how the ASCs mediate their effect, they have been shown to have immunomodulatory and proangiogenic properties, the ability to promote keratinocyte and fibroblast growth, as well as ability to reduce tissue scarring [8,9,10,11,12,13]. However, less is known about the putative effect of ASCs on the ECM of the chronic wounds. Consequently, in this review we will outline the role of ECM in wound healing, describe what is known regarding ASCs’ effect on ECM, and speculate on how ASC-derived ECM may be exploited in novel wound healing therapies.

## 2. The ECM of the Skin

In human skin, the ECM contains both fibrous proteins and ground substance. The fibrous proteins comprise collagens, elastin, and fibronectin, and provide a three-dimensional scaffold upon which both individual cells and the vascular network are supported or anchored. The most abundant fibrous protein in the human skin is collagen I, with collagen III and collagen V representing only minor proportions of the total collagen [14]. During pathological conditions such as scar formation, the composition and structure of collagen fibers are altered [15]. The ground substance of the ECM contains proteoglycans and glycosaminoglycans, and surround the fibrous proteins as a jelly-like substance which provides hydration to the skin due to the strong hydrophilic characteristics.

Initially, ECM was thought to function only as structural support for the cells; however, it has become clear that the ECM plays a pivotal role in the regulation of cell behavior both under normal conditions and during wound healing [16,17]. The ECM regulates cell behavior through molecular signaling primarily mediated by integrins (a family of cell surface receptors), and it has been shown that these signals are involved in determining whether the cells proliferate, differentiate, or undergo apoptosis [18]. Among the resident skin cells that express integrins—and thus may be subjected to modulation by the ECM—are fibroblasts and keratinocytes [19]. In addition, proteins in the ECM modulate the activity of growth factors and cytokines such as platelet-derived growth factor (PDGF) and transforming growth factor-β (TGF-β), produced by activated platelets and macrophages, respectively [20,21]. Thus, the ECM functions as a reservoir by protecting the growth factors from degradation and controlling their release [22].

ECM homeostasis is partly controlled by the activity of matrix metalloproteinases (MMPs) and their counterpart, tissue inhibitors of metalloproteinases (TIMPs). The MMPs are mainly secreted by keratinocytes, fibroblasts, and endothelial cells [23], and TIMPS by—among others—mesenchymal stem cells (MSCs), keratinocytes, and fibroblasts [24,25]. Thus, the balance between MMPs and TIMPs is important for ECM remodeling, cell signaling, and cell migration [26], and it has been suggested that a high MMP/TIMP ratio could be a biomarker of non-healing wounds [27].

## 3. Role of ECM for Wound Healing

**Figure 1:**
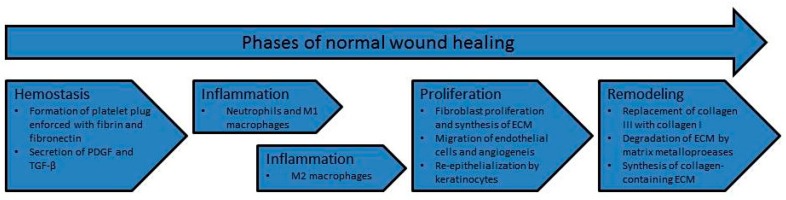
The phases of normal wound healing. Wound healing normally progresses through a tightly orchestrated process that is usually described as having four overlapping phases. During hemostasis, a platelet plug is formed and growth factors are secreted. The inflammatory phase has two stages. The initial stage, where neutrophils and pro-inflammatory M1 macrophages prevail, and a second stage characterized by the presence of anti-inflammatory M2 macrophages. During the proliferation phase, fibroblasts proliferate and synthesize extracellular matrix (ECM), new vessels are formed, and keratinocytes re-epithelialize the surface of the wound. In the final remodeling stage, the composition of the ECM is altered through degradation and resynthesis. PDGF: platelet-derived growth factor; TGF-β: transforming growth factor-β.

Acute wounds normally heal in four overlapping phases: hemostasis, inflammation, proliferation, and remodeling (Figure 1) [17,28,29]. Hemostasis occurs immediately after the injury, and is characterized by the activation and aggregation of platelets into the wounded area followed by the deposition of fibronectin and fibrin from the blood plasma. The activated platelets help initiate the inflammatory phase through the secretion of PDGF, which is important for the migration of macrophages and neutrophils to the wounded area [20], and TGF-β, which plays a major role in the transformation of monocytes to macrophages [21]. The stimulation of macrophages results in the development of polarized phenotypes termed classically activated (M1) macrophages that secrete pro-inflammatory cytokines and predominate during early wound healing and alternatively activated (M2) macrophages that are associated with a wound healing anti-inflammatory profile and which predominate in the later stages when inflammation abates and tissue undergoes remodeling [30,31].

During the proliferation phase of wound healing, fibroblasts migrate to the wounded area where they proliferate and initiate ECM synthesis [32]. The temporary matrix of fibrin and fibronectin is replaced by the collagen matrix, enriched in proteoglycans, glycosaminoglycans, and glycoproteins, forming a granulation tissue. Subsequently, the abundant extracellular matrix accumulates, supporting cell migration. In response to the newly-synthesized ECM, endothelial cells migrate into the wound and initiate the process of angiogenesis to restore the circulation in the damaged area [33]. The wound environment is characterized by low oxygen supply, regulating the process of angiogenesis through hypoxia-inducible factor-1 (HIF-1) [34]. Additionally, the secreted growth factors basic fibroblast growth factor (bFGF), TGF-β, and vascular endothelial growth factor (VEGF) stimulate the angiogenic activity [35]. Concurrently, keratinocytes migrate from the basement membrane towards the wound edge and close the wound. The migration of keratinocytes is dependent on basement membrane degradation, facilitated by MMPs [36].

In the remodeling phase, fibroblasts transform into myofibroblasts and contract the wound area [37]. Remodeling of the granulation tissue is characterized by the synthesis and breakdown of collagen, regulated by the MMPs and TIMPs [38].

**Figure 2:**
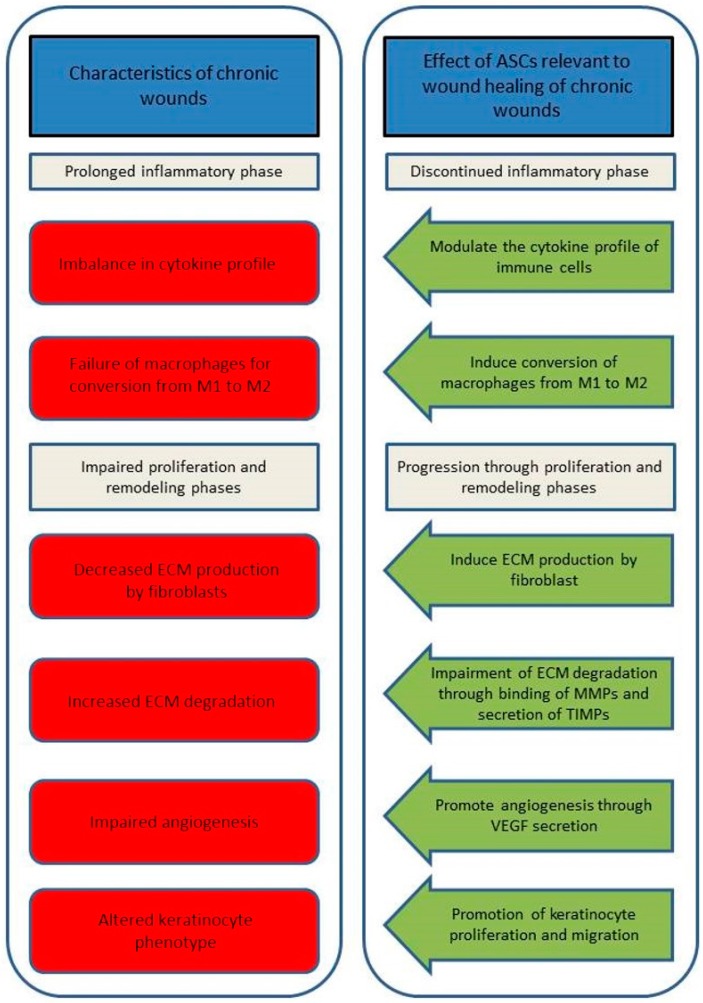
Characteristics of chronic wounds and the relevant regenerative effects of ASCs on these. Chronic wounds appear to unable to progress from the inflammatory phase of normal wound healing and to have impaired proliferation and remodeling phases (**left panel**). The ASCs have several regenerative characteristics that may lead to the wound progression from the inflammatory phase and through the proliferation and remodeling phases (**right panel**). ASC: adipose tissue-derived stem/stromal cells; MMP: matrix metalloproteinase; TIMP: tissue inhibitor of metalloproteinase; VEGF: vascular endothelial growth factor.

When the normal progression through the different phases of wound healing is perturbed as described above, the wounds may become chronic. It appears that non-healing wounds remain in a transition state between the inflammation and proliferation phases and proliferative and remodeling phases become impaired [39] (Figure 2, left panel). It is not clear what causes the prolonged inflammation; however, macrophages in chronic wounds fail to switch from the pro-inflammatory M1 to the anti-inflammatory M2 phenotype [40].

Furthermore, in mouse models of wound healing, there was a correlation between the presence of M2 macrophages, the resolution of inflammation, and wound healing, suggesting an important role of the polarization from M1 to M2 macrophages during the process of wound healing [41,42]. Interestingly, a switch in phenotype towards a more anti-inflammatory or pro-healing type has also been documented for Th1/Th2 cells and MSCs [43,44], which are possibly recruited to the site of injury from the bone marrow [45].

As wounds become chronic, the ECM homeostasis of the wound area is affected. Indeed, chronic wound fibroblasts are unresponsive to the stimulatory effect of TGF-β on collagen synthesis when compared to normal skin fibroblasts [46]. In addition, proteolytic enzymes involved in ECM degradation are dysregulated in chronic wounds, with increased expression of MMP-1, MMP-2, MMP-3, MMP-8, and MMP-9 [47,48] and decreased expression of the MMP inhibitor TIMP-2, leading to excessive proteolysis of the ECM [48]. As the balance between ECM synthesis and degradation is impaired, the ECM becomes dysfunctional in terms of supporting cell migration and proliferation as well as angiogenesis [49]. 

## 4. Using Adipose Stem Cells to Treat Chronic Wounds

The conventional treatment strategy for wound healing is based on wound bed preparation using tissue debridement, antibiotics, anti-inflammatory drugs, the restoration of moisture balance, and/or acceleration of epithelization by growth factor therapy [50,51]. Although these treatment options accelerate the wound healing process in many cases, many wounds are resistant to the current treatment options and more efficient methods are needed [49].

Recently, stem cell therapy has emerged as a novel approach for chronic wound healing. So far, most data is from studies using bone marrow-derived MSCs (BM-MSCs). However, as ASCs and BM-MSCs share numerous biological properties, much of the knowledge regarding BM-MSCs can be directly applied to the ASCs [52]. It has also become apparent the vastly higher numbers of ASCs than BM-MSCs can be obtained in a short time frame [52]. Thus, as procedures for the isolation and expansion of ASCs for clinical use have been optimized [53], ASCs are emerging as the most promising candidate for stem cell-based therapies for chronic wounds.

In the chronic wound environment, in vitro and in vivo studies suggest that the ASCs may be able to discontinue the prolonged inflammation phase and restore the progression through the phases of proliferation and remodeling (Figure 2, right panel). In terms of effects on the inflammatory processes, it is well known that ASCs may influence the functional characteristics and cytokine profile of T-, B-, and dendritic cells [54,55,56]. Notably, ASCs have also been shown to be able to induce a conversion of the macrophage phenotype from the pro-inflammatory M1 associated with chronic wounds to the anti-inflammatory and wound healing M2 phenotype [57,58]. During the proliferation phase, secreted factors from ASCs enhance several fibroblast characteristics, such as cell proliferation, migration and, importantly, the synthesis of collagen and other ECM proteins [59,60,61]. Furthermore, ASCs have been demonstrated to inhibit ECM degradation through the increased binding of MMPs and secretion of TIMPs [24]. The ability of ASCs to promote new vessel growth is also relevant to wound healing [62]. Finally, in vitro studies suggest that ASCs may promote re-epithelialization through modulation of keratinocytes in terms of promoting their proliferation and migration, but more studies are needed to confirm if this also holds true for chronic wounds [11,63].

To potentiate the wound healing effects of ASCs, the possibility of pre-conditioning the cells during in vitro expansion prior to clinical use has been suggested. In particular, the use of hypoxic culture appears interesting, as several of the wound healing properties of ASCs appear to be enhanced [64,65]. Significantly, it was recently found that hypoxic culture of ASCs altered their expression profile of several proteins related to ECM structure and function [66]. However, more data is needed to determine if the hypoxic potentiation of the regenerative properties of the ASCs in vitro can be translated into an enhanced effect in vivo.

## 5. ECM-Based Scaffolds for Wound Therapy

An alternative approach to using cells for wound therapy is to use acellular ECM. Acellular ECM-based scaffolds derived from natural tissues have been successfully applied in various preclinical and clinical settings for the treatment of severe wounds. These natural scaffolds appear to mediate tissue regeneration through a process known as constructive remodeling, in which the diverse ECM components orchestrate a process of scarless tissue repair [67]. There are various commercially available ECM-derived materials that are routinely used for the treatment of burns and chronic wounds, including materials obtained by the decellularization of animal tissues, such as porcine or bovine skin [68,69], or from allogeneic human skin [70]. A more detailed review of the variety of decellularized ECM scaffolds that are currently available for clinical use can be found in the literature [71]. Despite the relatively high success rates associated with these materials, some issues may still appear, such as sustained inflammatory responses and incomplete healing due to poor integrity of the native ECM molecules after decellularization [72]. In addition, xenogeneic ECM components may cause adverse host immune responses, and there is a risk of pathogen transfer [73]. To avoid these risks and the limitations associated with the supply of allogeneic human tissues, cell cultures have recently emerged as viable alternatives for the fabrication of ECM scaffolds. Depending on the cell type used for ECM synthesis, it is possible to fabricate ECM scaffolds containing specific proteins and morphogens that appear during early tissue development and which are associated with enhanced wound healing [74]. In particular, matrices derived from stem cells have shown promise as scaffolds for various tissue engineering and regenerative medicine applications, including regeneration of cartilage [75], bone [76], and neural tissue [77]. Surprisingly, despite the beneficial properties of BM-MSCs or ASCs in the context of wound healing therapies, little is known regarding the use of stem cell ECM for wound healing applications. In this context, MSCs may possess a relative advantage over terminally differentiated skin fibroblasts, as they have shown an increased capacity to synthesize proteins involved in extracellular matrix, morphogenesis, and development [78,79]. The predominant upregulation of genes such as fibronectin (*FN1*) and extracellular matrix protein 2 (*ECM2*) found in MSCs suggests that the ECM derived from these cells may enhance wound healing by promoting matrix deposition and cell adhesion [78]. Accordingly, comprehensive proteomic analysis of ECM derived from MSCs has revealed an enrichment of structural proteins, including collagen I, VI, and XII, which together with an increased presence of MMPs indicates a highly dynamic matrix turnover [79]. Furthermore, MSC-derived ECM is also enriched in proteoglycans such as perlecan and hyaluronan, and glycoproteins such as fibronectin, tenascin-C, fibulin-1, and thrombospondin-1 [79]. Overall, these components of the ECM may contribute to the different phases of wound healing by supporting integrin-mediated cell adhesion and signaling, cell migration, and proliferation. In addition, decellularized stem cell ECM has demonstrated a significant angiogenic potential, which has been evidenced through the activation of endothelial cells [80]. An additional advantage of using stem cell cultures is the possibility of microenvironmental preconditioning of the cells during the fabrication process to tailor specific biological or biophysical functionalities in the scaffold that may promote wound healing [81]. In ASCs, in vitro ECM production and assembly has been shown to be controlled by mechanical and topographical cues from the microenvironment [82,83].

Decellularized ECM-scaffolds may be also used as platforms for cell delivery. It has been hypothesized that ASCs might have a better survival rate and reduce scar formation when administrated in combination with ECM-components [84]. Such a co-delivery could be implemented either using a patch of ECM seeded with ASCs [84] or delivering the ASCs in a fibrin spray glue.

In summary, although fabrication of ECM scaffolds using ASC cultures or co-delivery of ASCs and ECM represent novel concepts that may offer several comparative advantages for wound healing applications, the knowledge in this field is still scarce, and more efforts are needed to further develop these approaches into a clinical reality.

## Abbreviations

ASC	Adipose stem cell/adipose stromal cell
bFGF	Basic fibroblast growth factor
BM-MSC	Bone marrow-derived mesenchymal stem cell
ECM	Extracellular matrix
HIF-1	Hypoxia-inducible factor 1
MSC	Mesenchymal stem cell
MMP	Matrix metalloprotease
PDGF	Platelet-derived growth factor
TGF-β	Transforming growth factor-beta
TIMP	Tissue inhibitor of metalloproteases
VEGF	Vascular endothelial growth factor
